# Supplementary material for: Satellite data indicates recent Arctic peatland expansion with warming
Source: Commun Earth Environ. 2025 Jun 19;6(1):461. doi: 10.1038/s43247-025-02375-1 (PMC12178905; doi:10.1038/s43247-025-02375-1)
Supplement: Supplementary file 2 — Supplementary information [file 43247_2025_2375_MOESM2_ESM.pdf]

Supplementary material "Satellite data indicates recent Arctic peatland expansion with warming"

Crichton *et al*

Supplementary Materials

Content

A. Site description and field photos

B. Remote sensing methods

C. Additional Carbon-14 evidence

SUPPLEMENTARY METHODS

A. Site description and field photos

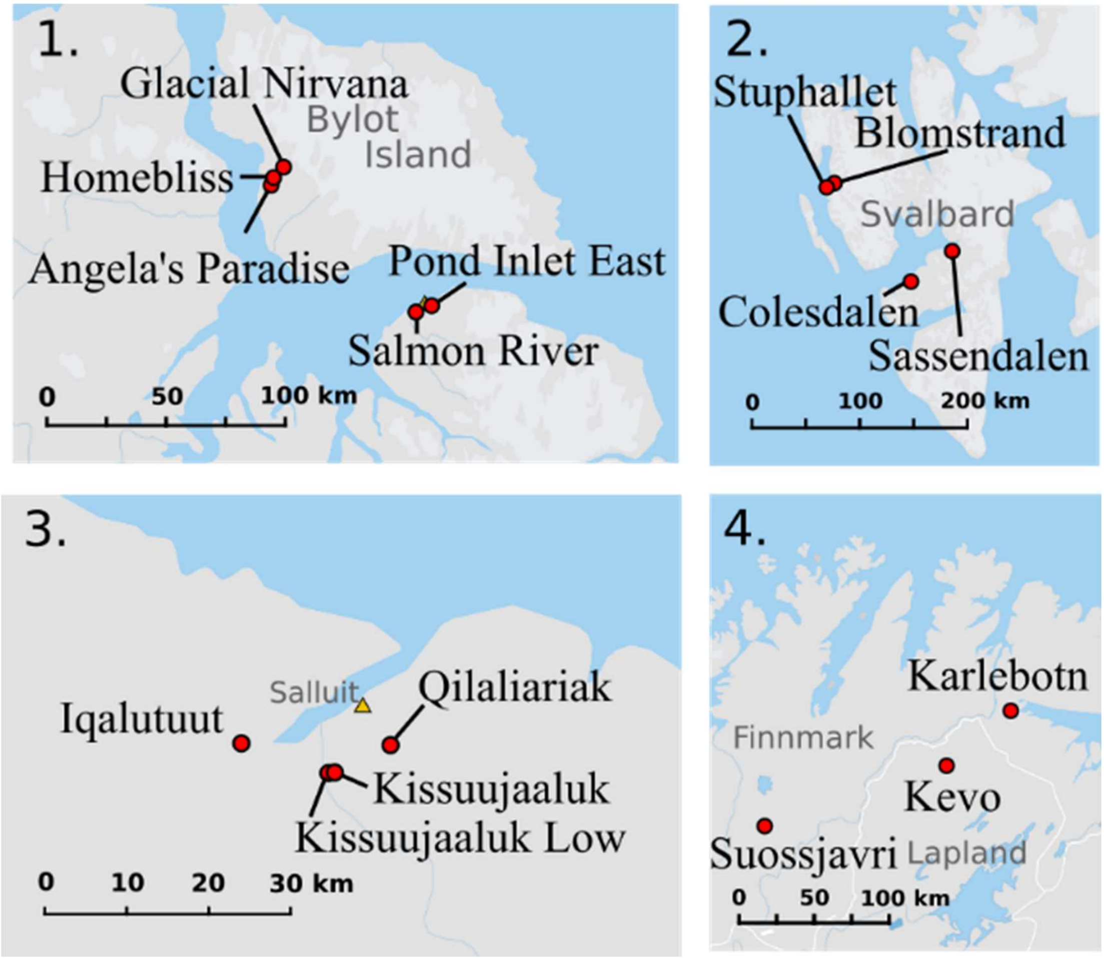

Fig S1, Overview maps showing the same of each site and their location. Coordinates for the sites and their transects are available in supplementary data file ICAAP_RS_areas_and_points.xlsx

Table S1, General sites descriptions per region and site

Region	General Description	Sites
High Arctic Canada, continuous permafrost zone	**Bylot Island** - Arctic tundra, with permafrost processes maintaining localised high moisture conditions where wetlands have developed. Sites are located within a glacial valley and plain, and have some patterned ground in places. Bylot island is a protected area, with many bird colonies. Plant communities at the field sites are dominated by Sphagnum mosses and herbaceous plants, and characterised as low centred polygon mires.<br><br>**Pond Inlet** sites - located across the water from Bylot Island, Arctic tundra drier in appearance than Bylot. Sites located on more exposed higher ground, not glacial valleys. Plant communities are mosses, sedges, grasses and some dwarf shrubs.	Glacial Nirvana (Bylot), Homebliss (Bylot), Angela's Paradise (Bylot)<br><br>Pond Inlet East (Pond Inlet, Salmon River (Pond Inlet)
High Arctic Europe, continuous permafrost zone	**Svalbard**<br><br>Near Ny Alesund in the Kongsfjorded, Blomstrand and Stuphallet sites are located below higher ground/cliffs (with possible bird nesting), very close to the coastline. Plant communities are mostly mosses, some grasses and few vascular plants, no shrubs.<br><br>Further south, located in the Isfjorden, Colesdalen and Sassendalen are larger expanses of vegetated areas. Both within post glacial valleys, and towards the coastline. Plant communities also mosses, grasses and some vascular plants.	Blomstrand, Stuphallet, Colesdalen, Sassendalen
Lower Arctic Canada, continuous permafrost zone	**Salluit**<br><br>Located around a coastal inlet, these sites are shallow coastal peatlands. Qilaliariak is on higher ground further from the coast, with the peat forming around a stream in a valley. All other sites are nearer the river/inlet coast. Plant communities are mostly mosses, some sedges and some grasses.	Peat Qilaliariak, Iqalutuut, Kissuujaaluk, Kissuujaaluk low
Low Arctic Europe, discontinuous and sporadic permafrost	**Lapland**<br><br>Located more inland, Lapland sites are characterised as degraded palsa mire and/or subarctic peatland. Plant communities include mosses, small shrubs, lichens, sedges, grasses and some trees on the edges of peatlands.	Karlebotn, Kevo, Suossjavri

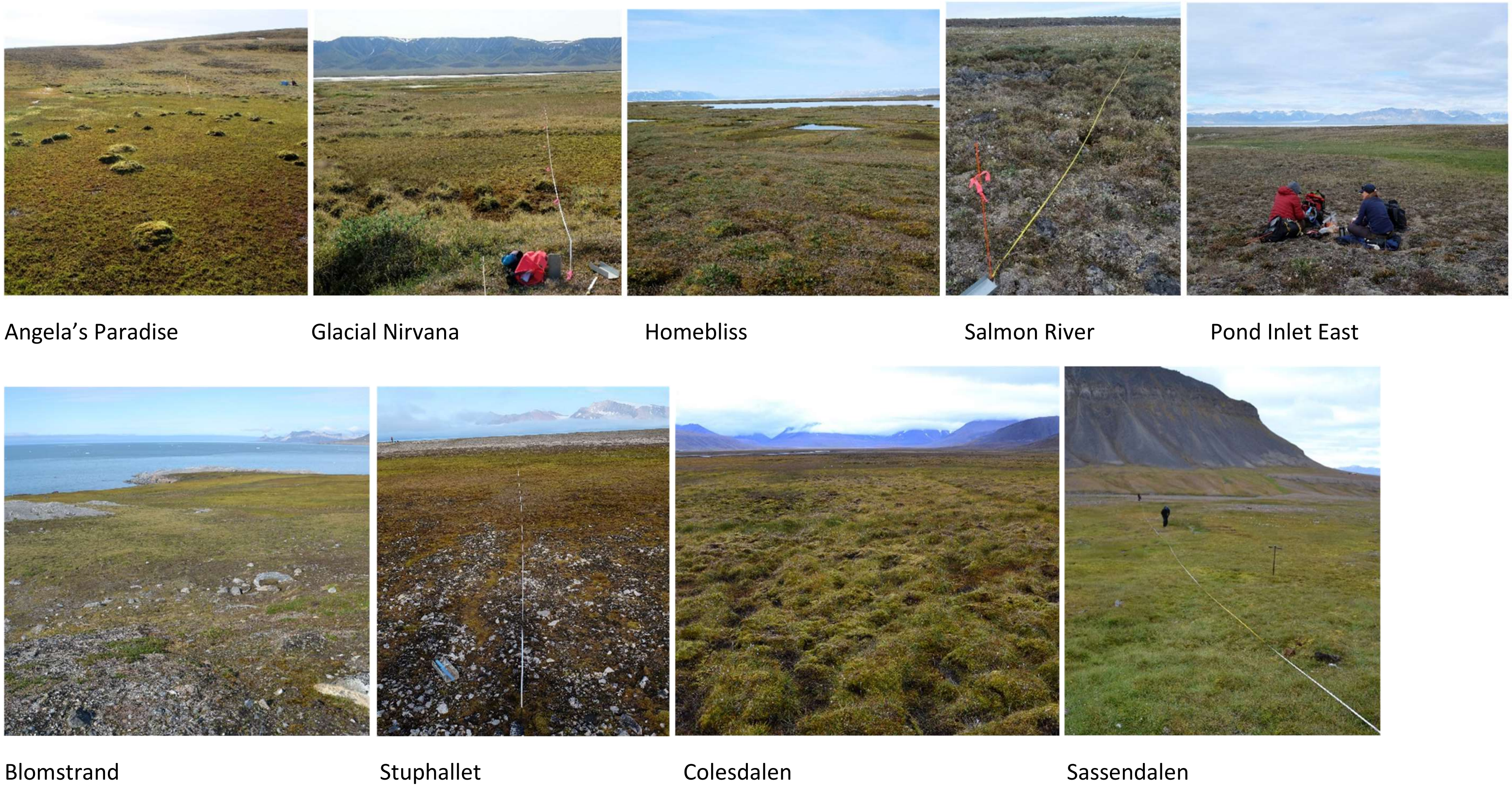

Fig S2, Field site photos for High Arctic locations (ICAAP team, Bylot and pond Inlet sites summer 2022, Svalbard sites summer 2019)

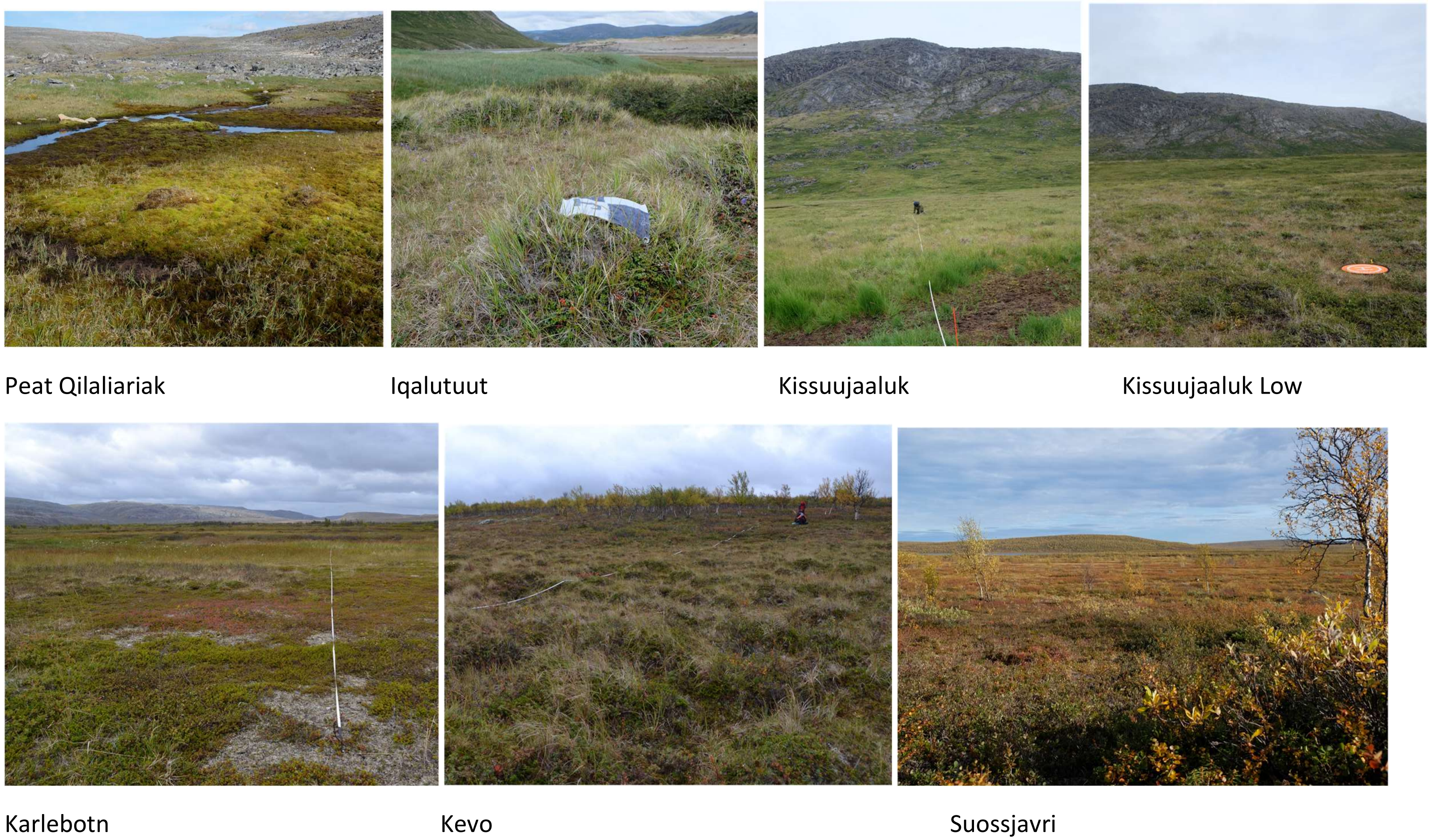

Fig S3, Field site photos for Lower Arctic locations,(ICAAP team, Salluit sites summer 2022, Lapland sites summer 2019)

B. Remote sensing methods

The Landsat data was extracted using Google Earth Engine. Satellite images used were surface reflectance data from Landsats 5 and 7, collection 2 level 2 tier 1 data. We use surface reflectance data even for these high latitudes because our analysis is only based on band ratios (as NDVI and NDMI). The rationale for discounting Landsat 8 data is explained in the main text methods section.

<u>Peat areas</u>

For the peat-areas, an area was defined using the satellite visible bands image and NDVI values that encompassed the transects (see Figs S4 to S7), excluded large water bodies and areas of very different land cover (such as, noteably higher topography, very different NDVI characteristics to the transect). The size of this area was a minimum of 150 pixels. Peat areas were also adjusted to avoid blank pixels caused due to the L7 SLC failure where possible. For each period (early or late period), images were pre-selected using a cloud filter, and day-of-year filter to have only peak-summer values. Any cloudy pixels (as identified using the QA_PIXEL band) in remaining images were masked, table 1 shows the number of images used for each. NDVI and NDMI value were calculated for all remaining pixels for all images in the period, and the maximum value was selected to represent the period. A further mask (on top of the cloud mask) was applied to remove outlier values (where the index was <100, so clearly erroneous), and this combined mask was applied to both periods to ensure we only compare pixels with valid values in each period. This masked max-value layer was used to create pixel-count histograms for each period (shown in full in Fig S8). The year 1992 was removed from the analysis for the Suossjavri site, due to anomalously-high values in NDMI (so we consider it to be an outlier). The “peat-area” is not to be understood as the actual edges of the peatlands, only a representative set of pixels that can be used to inform and compare what is happening in this wider area to what is seen along the transects.

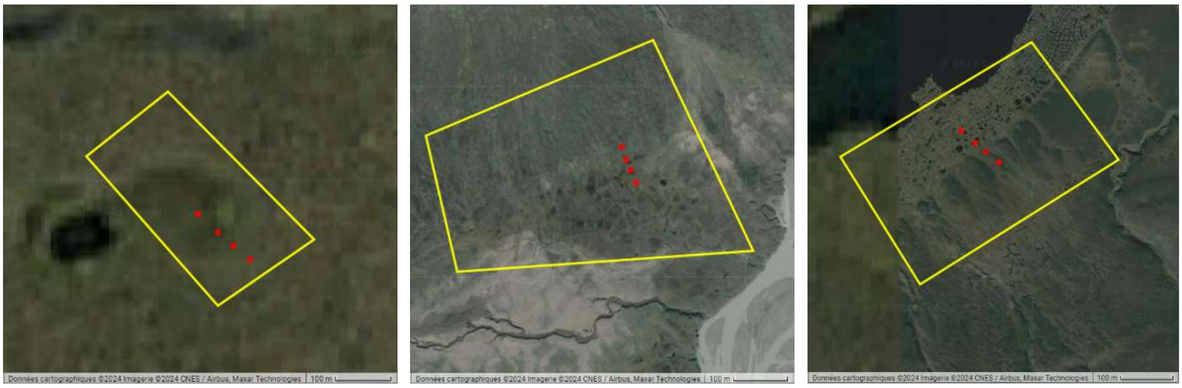

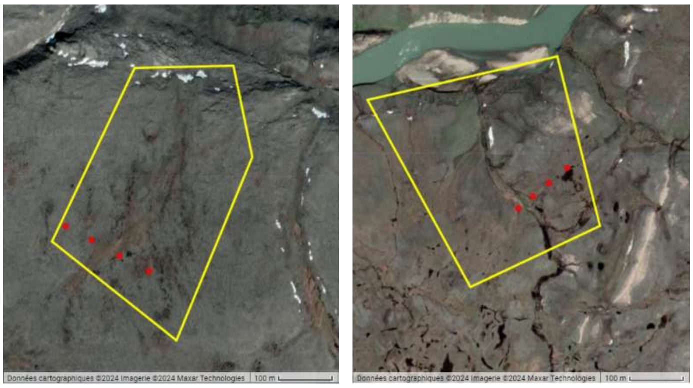

Fig S4, peat-area (yellow) and transect points (red). High Arctic Canada: Angela’s Paradise, Glacial Nirvana, Homebliss, Pond Inlet East, Salmon River. Images created in Google Earth Engine.

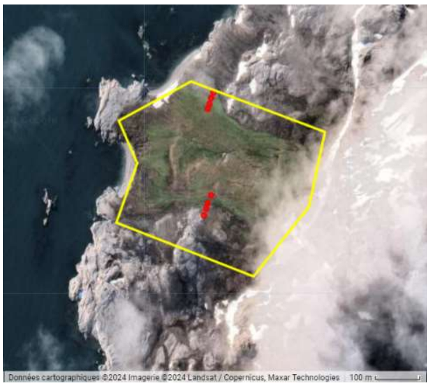
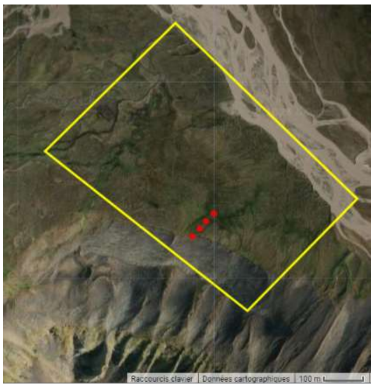
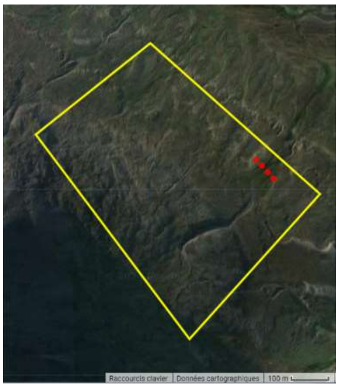
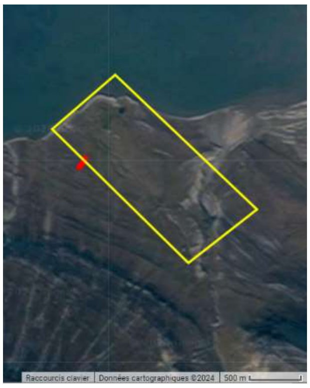
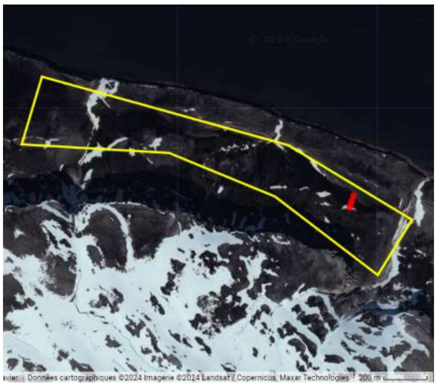

Fig S5, peat-area (yellow) and transect points (red). High Arctic Europe: Blomstrand, Colesdalen 1, Colesdalen 2, Sassendalen, Stuphallet. Images created in Google Earth Engine.

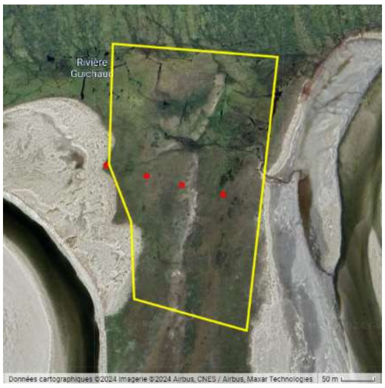
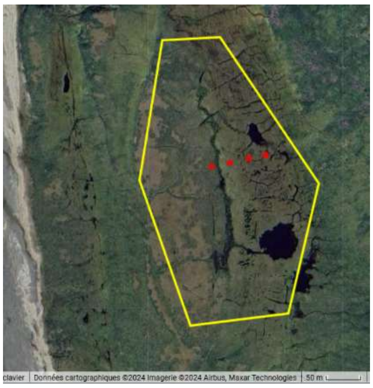
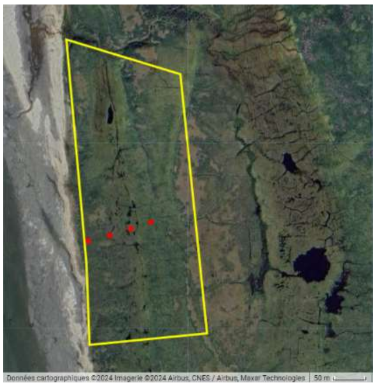
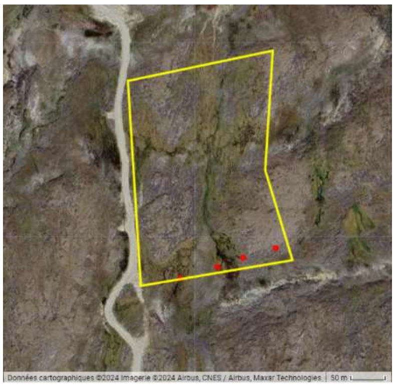

Fig S6, peat-area (yellow) and transect points (red). Arctic Canada: Iqalutuut, Kissuujaaluuk, Kissuujaaluuk Low, Peat Qilaliariak. Images created in Google Earth Engine.

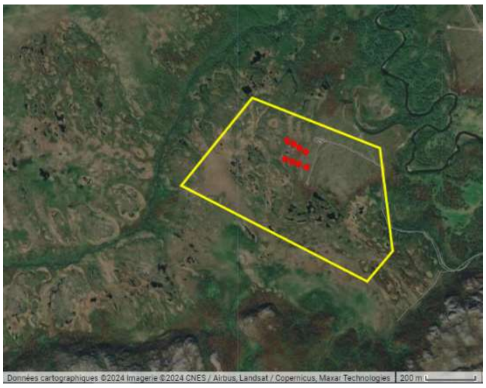

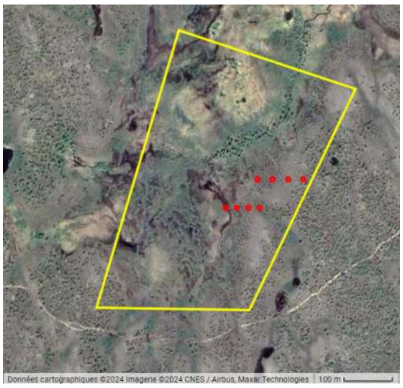

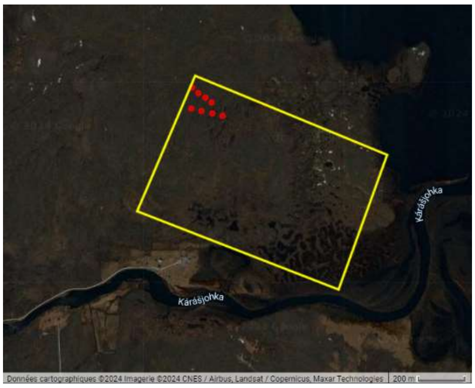

Fig S7, peat-area (yellow) and transect points (red). Low Arctic Europe: Karlebotn, Kevo, Suossjavri. Images created in Google Earth Engine.

<u>Transects</u>

The edge-to-peat transects are based on field-site-visit transects that were defined to extract peat monoliths for later lab study. These transects varied in length in the field, and for almost all it was necessary to extend; Landsat pixel size is 30m x 30m, so a transect length in the order of 30m had to be extended (otherwise all data along the transect could be identical). In order to do this, we inspected the peak-summer NDVI values, (generally) extending towards the higher values to go further in-peat, or lower NDVI values further outside the field-transect edge. Some of the sites had quite small/sporadic peat cover (on the scale of the Landsat pixel), so transects may not always represent a linear path from edge-to-peat. This is visible in Figs S9 showing the drone images for the transect points. For example, Peat Qilaliariak is characterised by a shallow small valley topography, where peat is forming, with greatest peat cover in point 3. Salmon river, similarly, has a depression where peat is forming, with more sporadic vegetation cover either side. Due to this uncertainty, itself due to Landsat pixel size, we do not focus extensively in the text on the specific difference between changes per-point, but rather focus on changes seen in the edge pixels, and compare this to what we see along other points on the transect.

Point data for these 4 transect points were extracted using Google Earth Engine. For both Landsat 5 and Landsat 7 all data between day 160 and 280 of the year was extracted for all bands. This data was loaded onto excel sheets and further analysis and filtering was applied here. Using the QA_PIXEL band any points identified as cloud or cloud shadow was excluded. Any clear outliers (identified by plotting the data, and inspection of data values) were manually excluded. The data was put in to a pivot table to find the mean yearly peak-summer NDVI and NDMI values for each point. The day of the year for peak-summer for each site is 185 to 215 for Svalbard; day 190 to 210 for Lapland; day 190 to 220 for Bylot Island and Pond Inlet; day 200 to 230 for Salluit. These values were set by inspection of the NDVI variability over the summer period per site. This yearly-mean data was then grouped in to early or late periods; for all sites the early period is 1985 to 1995, for the low Arctic the

late period is 2010 to 2020, for the high Arctic the late period is 2005 to 2015. This difference in late period years is due to cloud cover and image availability.

Table S2, Number of images used for the peat-area based distributions and cloud cover pre-filter (as % over which scenes were excluded) for each site

Region	Site name	cloud	img early	img late	notes
high Arctic Canada	Angela's Paradise	10	9	10	
	Glacial Nirvana	10	9	10	
	Homebliss	10	9	10	
	Pond Inlet East	10	4	11	
	Salmon River	10	5	12	
Svalbard	Blomstrand	10	1	2	very low number of images at 10% cloud filter, cloud cover issue generally
	Stuphallet	80	15	23	cloud filter (for full scene) increased to get more images of good quality
	Colesdalen 1	30	1	7	cloud filter (for full scene) increased to get images of good quality
	Colesdalen 2	30	3	7	
	Sassendalen	20	1	3	cloud cover limits number of early images
Salluit	Iqalutuut	10	8	5	
	Kissuujaaluk	10	9	5	
	Kissuujaaluk Low	10	9	5	
	Qilaliariak	10	10	7	
Lapland	Karlebotn	80	35	34	cloudy pixels and SLC failure mean many images required (and cloud filter at 80)
	Kevo	80	34	35	cloudy pixels and SLC failure mean many images required (and cloud filter at 80)
	Suossjavri	25	4	7	

Table S3, transect length calculated from end coordinates using Haversine function

**Site**	**Transects extension notes**	**Field transect length (m)**	**RS data transect length (m)**
Angela's Paradise	No drone data available, transect extended in both directions	36	125
Glacial Nirvana	No drone data available, transect extended in both directions	18	71
Homebliss	No drone data available, transect extended in both directions	27	94
Pond Inlet East	This is an edge to edge transect, and small peat-area, the transect is extended in both directions, peak NDVI actually falls in point 3	39	178
Salmon River	Edge-to-edge transect on small peat-area, transect extended to low NDVI side (lower NDVI present on both sides)	36	118
Blomstrand 1	Transect extended in to the peat area	29	49
Blomstrand 2	Transect extended in to the peat area	12	38
Stuphallet	Edge-to-edge transect, highest NDVI in point 3. Transect not extended	62	58
Colesdalen 1	Transect extended in to the peat area	40	81
Colesdalen 2	A smaller/non-constant peat-area, transect extended in both directions	23	70
Sassendalen	A smaller/non-constant peat-area, transect extended in both directions	51	99

Site	Transects extension notes	Field transect length (m)	RS data transect length (m)
Iqalutuut	Transect extended in to the peat area	46	183
Kissuujaaluk	Transect extended in to the peat area	20	84
Kissuujaaluk Low	Non-constant/sporadic peat areas towards water, transect extended in both directions	16	101
Peat Qililiariak	A smaller/non-constant peat-area, transect extended in both directions	37	148
Karlebotn 1	Transect extended in to the peat area	28	93
Karlebotn 2	Transect extended in to the peat area	28	87
Kevo 1	Transect extended in to the peat area	47	71
Kevo 2	Transect extended in both directions, but more towards lower NDVI	49	96
Suossjavri 1	Transect extended in to the peat area	71	105
Suossjavri 2	Transect extended in to the peat area	92	134

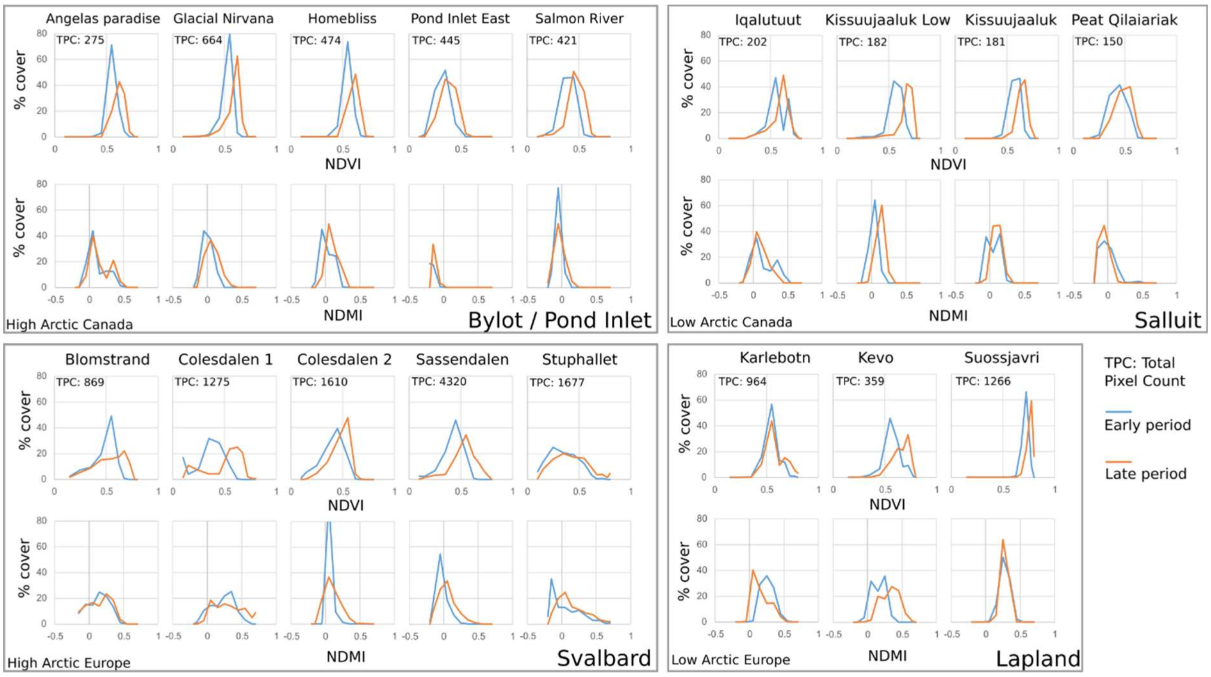

Fig S8, distributions of NDVI and NDMI for early (shown in blue) and late (shown in orange) periods for all peat areas, the change between these two distributions is shown in figure 3C in the main text.

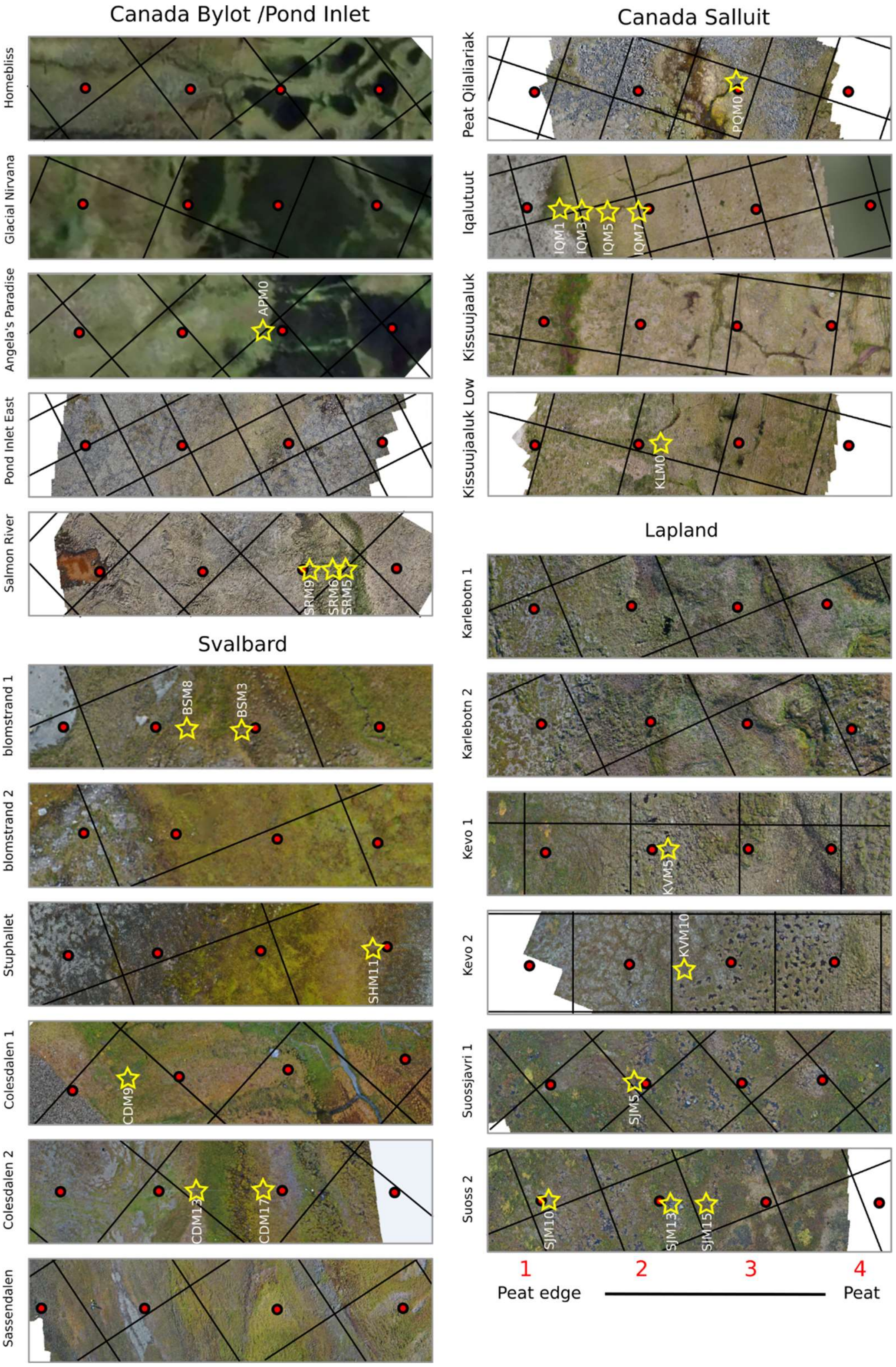

Fig S9, Overview of transects used in this study. Images from the drone, or from Mapcarta (for Bylot sites, where we do not have drone data). Red dots are points where Landsat data is extracted, all shown in transect order from peat edge towards peat centre (point 1 to point 4). Lines overlaid show the Landsat 8 grid orientation and size (each square is 30m by 30m) – Landsat 5 and 7 grid do not always correspond, but here indicates the effect of satellite pixel size. Yellow stars show the location of monolith cores with 14C dates reported in figure S10. Bylot sites show wet-centred polygons, which were almost entirely filled-in by the time of our field visits (2022).

C. Additional chronological ^{14}C evidence

Our study uses the very recent past as a reference period that determines the edges of the peatland, according to field site visits. These site visits were carried out as part of the ICAAP project, which aims to combine various lines of evidence to consider whether there is an increased carbon accumulation in Arctic peatlands. In the current study we use remote sensing data from the Landsat family of satellites to consider these same sites' characteristics from up to ~40 years ago. This method allows us to consider changes on the land surface, which show apparent recent expansion of peat-forming plant communities. As an additional line of evidence, we have the benefit of some preliminary below-surface data in the form of ^{14}C dating. With this data we can show that these sites have very likely been accumulating peat since the start of the Landsat timeseries we have studied in the main text (so, from 1985).

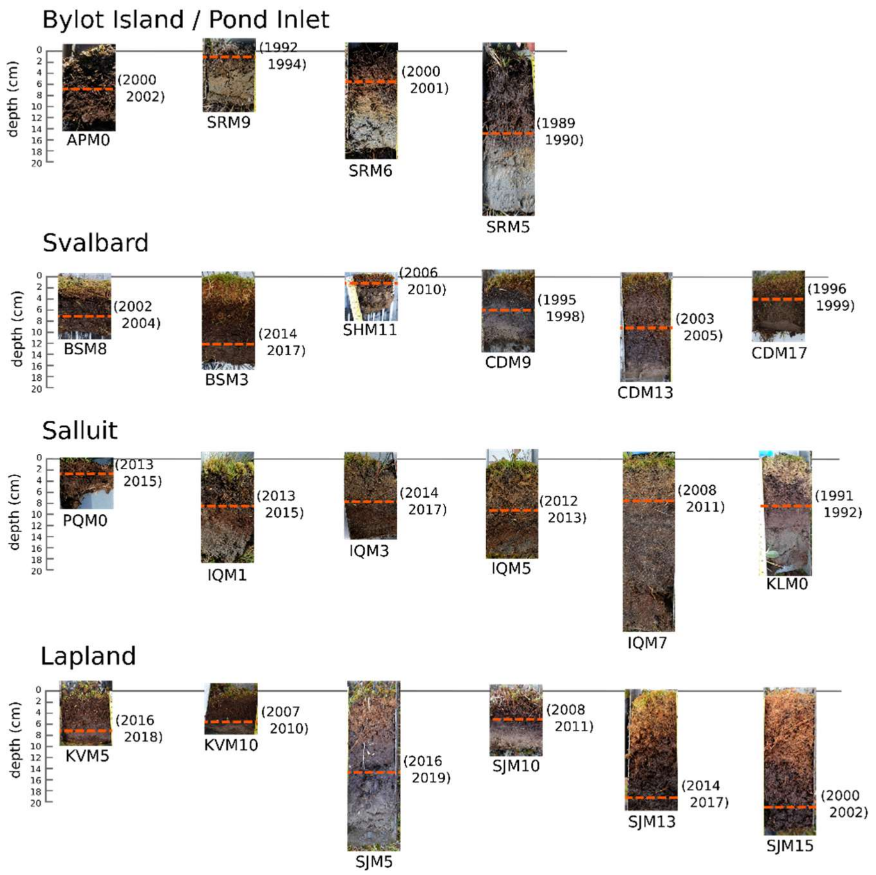

Fig S10, Selection of monolith cores collected as a sister study, with those basal-peat ^{14}C calibrated ages falling within the time period of interest (1985 to 2020). Peat growth has been continuing at these sites over the last 39 years. Full ^{14}C results are given in table S4. Soils present below the basal-peat (red dashed line) are non-peat soils.

<u>Dating methodology</u>

"Basal peats were dated by ^{14}C analysis using primarily plant macrofossils, however where no suitable macrofossils were present, bulk peat samples were prepared. Any surface material, or material exposed on the outside of the core was removed in case of contact with modern material and contamination. Samples were sieved through a 250-micron sieve. Material >250 µm was transferred to a petri dish and floated in deionised water. The samples were analysed under a low-powered stereo microscope and visually assessed for suitable above ground material for dating.

Picked plant macrofossils were transferred to a glass vial with deionised water and a couple of drops of HCL. These were sent to the University of Ottawa, Canada (Svalbard samples) or Queen University Belfast (Lapland and Canada samples), for radiocarbon dating via accelerated mass spectrometry (AMS)… Each ^{14}C date was calibrated using the IntCal20 (Reimer et al., 2020) and NH1 post-bomb (Hua et al., 2013) radiocarbon calibration curves" (Handley et al *In Prep*).

<u>Implications for our study</u>

The soil samples sub-set we provide shows that peat carbon has been actively accumulating at our sites in the recent past. Our remotely sensed data shows that at the same time, the sites have been greening, in the centres and at the edges, and peak-summer moisture levels have remained fairly stable. The ^{14}C data shows that accumulation is not only occurring in the centre well-established peatland areas, but also at the edges, and very recently. Our satellite data shows an expansion of peat forming plant communities at edges, the below-ground ^{14}C data shows recent carbon accumulation in-peat and near peatland edges. From this we conclude that the peatlands have been expanding.

Table S4, Carbon-14 dates for cores, (location of cores is shown in Fig S9 relative to the transects). The low and high errors are given, along with Age (cal. year C.E.) and depth of the sample as shown in Fig S10.

ID	Lab ID	Region	Sites	$F^{14}C$	Error	Age (cal. year C.E.)	Depth (cm)
APM0	UBA-52355	Canada N Bylot	Angela's Paradise	1.091	0.0037	2000-2002 (89%), 1958-1958 (5%)	4.5
SRM5	UBA-53540	Canada N Pond Inlet	Salmon River	1.1716	0.0027	1989-1990 (85%)	10.5
SRM6	UBA-52367	Canada N Pond Inlet	Salmon River	1.098	0.0028	2000-2001 (77%)	5.5
SRM9	UBA-52533	Canada N Pond Inlet	Salmon River	1.1367	0.003	1992-1994 (87%)	0.5
BSM3	UOC-15023	Svalbard	Blomstrand	1.0279	0.0043	2014-2017 (84%), 1957-1957 (6%)	11.5
BSM8	UOC-15026	Svalbard	Blomstrand	1.0784	0.0044	2002-2004 (66%), 1957-1957 (24%)	6.5
SHM11	UOC-15044	Svalbard	Stuphallet	1.0578	0.0040	2006-2010 (94%)	1.5
CDM9	UOC-15047	Svalbard	Colesdalen	1.1165	0.0046	1995-1998 (91%)	5.5
CDM13	UBA-51692	Svalbard	Colesdalen	1.0783	0.0028	2003-2005 (69%), 1958-1958 (25%)	9.5
CDM17	UOC-15049	Svalbard	Colesdalen	1.1095	0.0046	1996-1999 (93%)	4.5
PQM0	UBA-52360	Salluit	Peat Qilaliariak	1.0345	0.003	2013-2015 (74%), 1957-1957 (19%)	2.5
IQM1	UBA-52534	Salluit	Iqalutuut Valley	1.0314	0.003	2013-2015 (83%), 1957-1957 (10%)	7.5
IQM3	UBA-52535	Salluit	Iqalutuut Valley	1.0249	0.0029	2014-2017 (87%), 1956-1956 (8%)	6.5
IQM5	UBA-52536	Salluit	Iqalutuut Valley	1.0388	0.003	2012-2013 (75%), 1957-1957 (18%)	8.5
IQM7	UBA-53537	Salluit	Iqalutuut Valley	1.0546	0.0029	2008-2011 (93%)	7.5
KLM0	UBA-52361	Salluit	Kissuujaaluk Low	1.1554	0.0031	1991-1992 (71%), 1959-1959 (20%)	8.5
KVM5	UBA-52155	Lapland	Kevo	1.0202	0.0029	2016-2018 (84%), 1956-1956 (10%)	7.5
KVM10	UBA-52157	Lapland	Kevo	1.0578	0.0034	2007-2010 (92%)	5.5
SJM5	UBA-51681	Lapland	Suossjavri	1.0186	0.0045	2016-2019 (82%), 1956-1956 (11%)	14.5
SJM10	UBA-52162	Lapland	Suossjavri	1.0523	0.0034	2008-2011 (88%)	4.5
SJM13	UBA-51686	Lapland	Suossjavri	1.0241	0.0041	2014-2017 (82%), 1956-1956 (8%)	19.5
SJM15	UBA-51687	Lapland	Suossjavri	1.0907	0.0028	2000-2002 (95%)	21.5

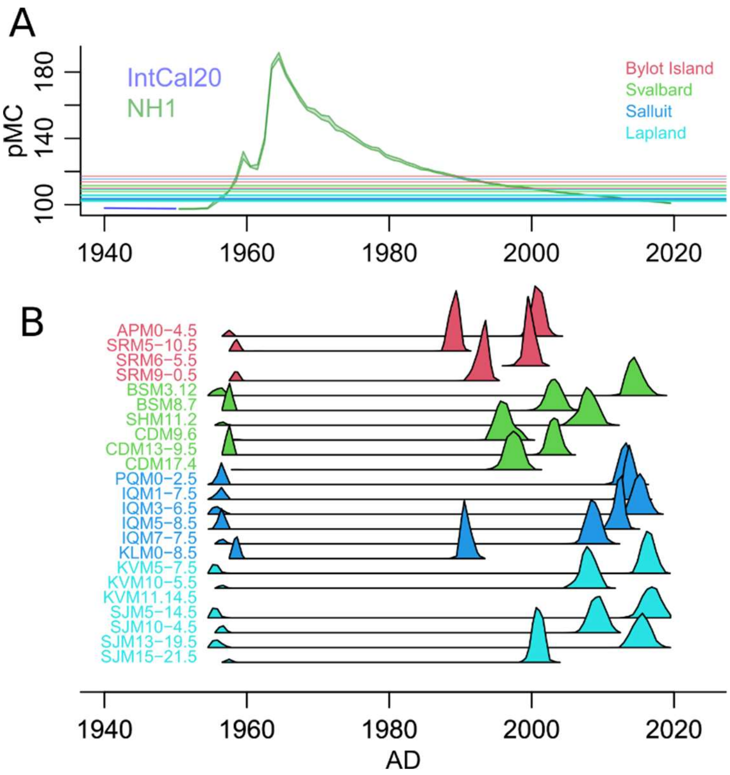

Fig S11, The (postbomb) calibration curve for the ^{14}C dating and the horizontal lines show the pMC ages of all the sites, colour-coded according to region (A), and the calibrated ages (B).

References

Hua, Q., Turnbull, J.C., Santos, G.M., Rakowski, A.Z., Ancapichún, S., De Pol-Holz, R., Hammer, S., Lehman, S.J., Levin, I., Miller, J.B. and Palmer, J.G., 2022. Atmospheric radiocarbon for the period 1950–2019. Radiocarbon, 64(4), pp.723-745. doi:10.1017/RDC.2021.95

Reimer, P.J., Austin, W.E.N., Bard, E., Bayliss, A., Blackwell, P.G., Ramsey, C.B., Butzin, M., Cheng, H., Edwards, R.L., Friedrich, M., Grootes, P.M., Guilderson, T.P., Hajdas, I., Heaton, T.J., Hogg, A.G., Hughen, K.A., Kromer, B., Manning, S.W., Muscheler, R., Palmer, J.G., Pearson, C., Plicht, J. van der, Reimer, R.W., Richards, D.A., Scott, E.M., Southon, J.R., Turney, C.S.M., Wacker, L., Adolphi, F., Büntgen, U., Capano, M., Fahrni, S.M., Fogtmann-Schulz, A., Friedrich, R., Köhler, P., Kudsk, S., Miyake, F., Olsen, J., Reinig, F., Sakamoto, M., Sookdeo, A., Talamo, S., 2020. The IntCal20 Northern Hemisphere Radiocarbon Age Calibration Curve (0–55 cal kBP). Radiocarbon 62, 725–757. https://doi.org/10.1017/RDC.2020.41
