# Supplementary material for: Satellite data indicates recent Arctic peatland expansion with warming
Source: Commun Earth Environ. 2025 Jun 19;6(1):461. doi: 10.1038/s43247-025-02375-1 (PMC12178905; doi:10.1038/s43247-025-02375-1)
Supplement: Supplementary file 3 — Description of additional supplementary files [file 43247_2025_2375_MOESM3_ESM.pdf]

Description of Additional Supplementary Files

File name: Supplementary Data
Description: site locations, processed RS pixel counts, processed RS transect values
